# Pleural empyema with gas formation caused by mixed infection of *Edwardsiella tarda* with *Streptococcus constellatus*

**DOI:** 10.1002/rcr2.913

**Published:** 2022-02-13

**Authors:** Yuki Ikematsu, Miiru Izumi, Tsuyoshi Ueno, Yuki Moriuchi, Mizuko Ose, Naotaka Noda, Makiko Hara, Junji Otsuka, Kentaro Wakamatsu, Masayuki Kawasaki

**Affiliations:** ^1^ Department of Respiratory Medicine National Hospital Organization Omuta National Hospital Fukuoka Japan

**Keywords:** anaerobes, *Edwardsiella tarda*, pleural empyema, *Streptococcus constellatus*, *Streptococcus milleri* group

## Abstract

*Edwardsiella tarda* is an anaerobic, gram‐negative rod bacterium associated with freshwater and marine life. Human *E. tarda* infections are rare, and most infections in humans cause gastroenteritis. Extraintestinal infections of *E. tarda* such as pleural empyema are particularly rare. A 72‐year‐old man was admitted with cough and purulent sputum. His medical history included periodontal disease and gastric cancer for which he had undergone total gastrectomy. Chest computed tomography showed left pleural effusion with foci of gas, and both *E. tarda* and *Streptococcus constellatus* were cultured from the pleural effusion. Thus, he was diagnosed with gas‐forming empyema. He was successfully treated with therapeutic thoracentesis and antibiotics. Our case suggests that a dietary habit of raw fish, undernutrition, gastrectomy and oral infection may be predisposing factors for empyema caused by *E. tarda*.

## Graphical abstract

*Edwardsiella tarda* is a motile, anaerobic, gram‐negative rod bacterium associated with freshwater and marine life. Previous reports have indicated that *E. tarda* is a rare human pathogen and that its most common clinical manifestation is gastroenteritis. We herein present the first case of pleural empyema with gas formation caused by mixed infection of *E. tarda* with *Streptococcus constellatus*, a member of the *Streptococcus milleri* group.

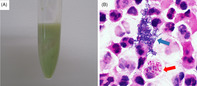

## INTRODUCTION

*Edwardsiella tarda* is a motile, anaerobic, gram‐negative rod bacterium associated with freshwater and marine life. Previous reports have indicated that *E. tarda* is a rare human pathogen and that its most common clinical manifestation is gastroenteritis.^1^ However, *E. tarda* infrequently causes extraintestinal infections such as septicaemia, meningitis, cholecystitis and liver abscess, which can become systemic and potentially lethal. Empyema caused by *E. tarda* is particularly rare,^2^ and gas production in such cases has not been previously reported. We herein present the first case of pleural empyema with gas formation caused by mixed infection of *E. tarda* with *Streptococcus constellatus*, a member of the *Streptococcus milleri* group.

## CASE REPORT

**FIGURE 1:**
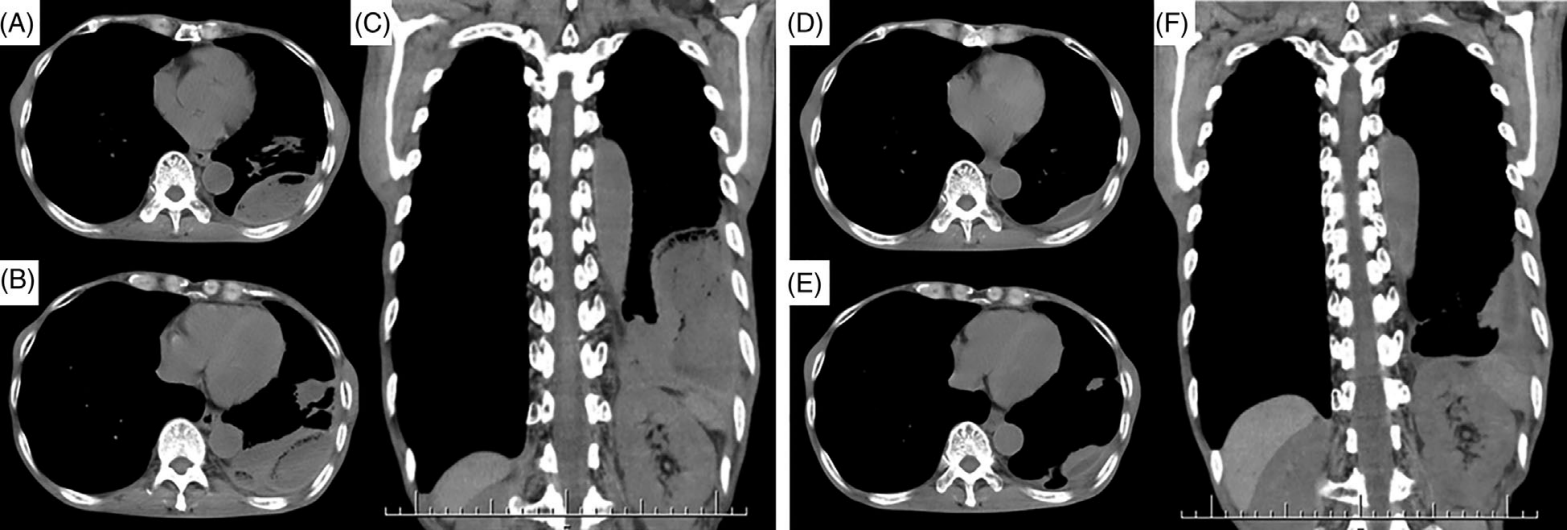
Computed tomography of images. (A–C) Before treatment, chest computed tomography (CT) revealed suspended air bubbles within the pleural effusion with septations in the left lower lung lobe. (D–F) After therapeutic thoracentesis and treatment with antibiotics for 4 weeks, CT showed marked shrinkage of the empyema, and no septations or gas foci were detected in the pleural effusion

**FIGURE 2:**
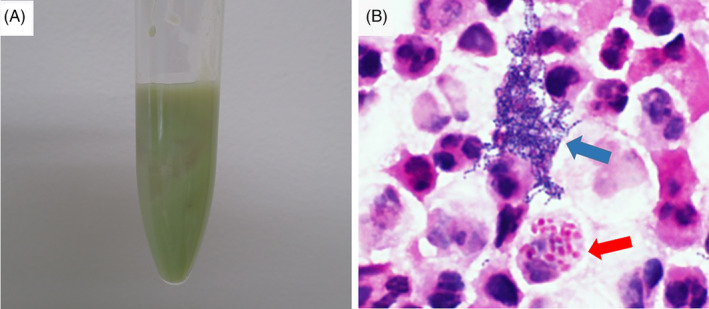
Pleural effusion analysis. (A) Left‐sided pleural effusion samples showed green purulent fluid. (B) Gram staining analysis of the pleural effusion under a microscope at 1000× magnification revealed many neutrophils and gram‐negative rods (red arrow) as well as gram‐positive cocci (blue arrow)

A 72‐year‐old Japanese man was admitted because of a 2‐week history of cough, purulent sputum and anorexia. The patient was thin (height, 157 cm; weight, 34.5 kg; and body mass index, 14.0 kg/m^2^), and his medical history included gastric cancer for which he had undergone total gastrectomy. He also had periodontal disease, but had discontinued the treatment for 2 years before the hospitalization. A dietary history revealed that he had eaten *Paraplagusia japonica*, a species of olive flounder, on a weekly basis. He had no history of taking immunosuppressive medication. His body temperature was 37.2°C and physical examination revealed coarse crepitations and reduced breath sounds in the left lower lung. Periodontal lesions were still detected. He had no gastrointestinal symptom or preceding history such as stomach pain, nausea and diarrhoea. Laboratory findings included a white blood cell count of 7100/μl with 82.5% neutrophils, C‐reactive protein concentration of 5.36 mg/dl, procalcitonin concentration of 0.07 ng/ml (reference range, ≤0.05 ng/ml) and low serum albumin concentration of 2.1 g/dl (reference range, 4.1–5.1 g/dl). Chest computed tomography (CT) showed an infiltration shadow and suspended air bubbles within the pleural effusion with septations in the left lower lung lobe (Figure 1A–C). Diagnostic thoracentesis was performed, and the pleural fluid was green, purulent and foul‐smelling (Figure 2A). The pleural fluid analysis was consistent with an exudative aetiology according to Light's criteria (Table 1). Microscopic examination revealed many neutrophils and gram‐negative rods as well as gram‐positive cocci. Furthermore, neutrophil‐mediated phagocytosis of gram‐negative rods was seen in the pleural effusion (Figure 2B). A culture of the fluid grew *E. tarda* and *S. constellatus*. However, these bacteria were not cultured from either spontaneous sputum or blood specimen. Finally, he was diagnosed with gas‐producing empyema caused by *E. tarda*, and treated with meropenem (2 g/day). In addition, therapeutic thoracentesis was performed, and a total of 105 ml of similarly appearing fluid was removed. Thereafter, the patient's clinical symptoms gradually improved and we found that the detected *E. tarda* was susceptible to meropenem we used and most other antibiotics including ampicillin, piperacillin, ceftriaxone, cefazoline and levofloxacin. Two weeks after the treatment, his C‐reactive protein concentration had decreased to 0.17 mg/dl, and de‐escalating antibiotic therapy of sulbactam/ampicillin was continued. Chest CT 4 weeks after the initial treatment showed marked shrinkage of the empyema, and no septations or gas foci were detected in the pleural effusion (Figure 1D–F).

**TABLE 1:** Pleural fluid analysis

Pleural fluid	
pH	6.0
Pleural fluid/serum protein (g/dl)	3.0/6.7
Glucose (mg/dl)	28
Pleural fluid/serum LDH	101,376/166
ADA	600.2
WBC count (/μl)	N/A
Neutrophils (%)	95
Eosinophils (%)	0
Monocytes (%)	1
Basophils (%)	0
Lymphocytes (%)	4

*Note*: WBC count in pleural fluid was not evaluated because of the high density of the fluid.

Abbreviations: ADA, adenosine deaminase; LDH, lactate dehydrogenase; N/A, not available; WBC, white blood cell.

## DISCUSSION

Risk factors for *E. tarda* infection include wounding in marine environments, exposure to infected reptiles and amphibians, a dietary habit of ingesting raw fish and immunodeficiencies.^1^ In our case, the patient had no history of contact with wild animals or aquatic environments; however, he had a dietary habit of eating a species of olive flounder that was a known representative host of *E. tarda*. Furthermore, his medical history revealed gastric cancer for which he had undergone total gastrectomy. Previous case studies have reported that gastrectomy may increase the risk of *E. tarda* bacteraemia due to reduction of gastric acid secretion.^3^ The patient also had a dental infection that served as a predisposing factor for pleural empyema. Interestingly, *E. tarda* was cultured only from the pleural effusion and not from the sputum. Therefore, *E. tarda* infection in the present case was considered to be foodborne, transmitted to the patient by ingestion of contaminated olive flounder with possible colonization in the oral cavity through periodontal disease followed by spread and establishment of purulent invasion in the pleural cavity.

Pleural empyema with gas formation is rare. *E. tarda* is an anaerobic bacterium that can produce hydrogen sulphide in laboratory media; however, gas‐forming infection caused by *E. tarda* is rare in the clinical setting. To the best of our knowledge, a case of empyema with gas formation caused by *E. tarda* infection has not been previously reported. In our patient, both *E. tarda* and *S. constellatus* were cultured from the pleural effusion. *S. constellatus* belongs to *S. milleri* group, which have been isolated from the mouth and nasopharynx. *S. constellatus* has low pathogenic potential and is less associated with severe infection including empyema than other *S. milleri* group subspecies such as *Streptococcus intermedius*.^4^ The present case showed neutrophil‐mediated phagocytosis of gram‐negative rods such as *E. tarda* in the pleural effusion; thus, *E. tarda* could plausibly have been responsible for causing gas‐producing empyema.

In conclusion, extraintestinal infections of *E. tarda* are rare; however, physicians should know that people with oral infections or poor nutrition who habitually ingest raw fish are at risk of empyema caused by this bacteria. Although *E. tarda* is sensitive to many antibiotics in vitro, *E. tarda* bacteraemia has a high mortality rate.^5^ Thus, immediate intervention of pleural drainage and appropriate antibiotic therapy should be performed in patients with empyema caused by *E. tarda*.

## CONFLICT OF INTEREST

None declared.

## AUTHOR CONTRIBUTION

Yuki Ikematsu was responsible for the patient's treatment and original draft preparation. Miiru Izumi was responsible for the treatment and contributed to the manuscript review. Tsuyoshi Ueno, Yuki Moriuchi, Mizuko Ose, Naotaka Noda, Makiko Hara, Junji Otsuka and Masayuki Kawasaki contributed to the writing of the final manuscript. Kentaro Wakamatsu was the chief investigator and was responsible for project administration.

## ETHICS STATEMENT

The authors declare that appropriate written informed consent was obtained for the publication of this manuscript and accompanying images.

## Data Availability

The data that support the findings of this study are available from the corresponding author upon reasonable request.
